# Preliminary study on the time-correlation changes in brain neurotransmitters of mice exposed to mushroom toxin ibotenic acid

**DOI:** 10.3389/fnins.2025.1561291

**Published:** 2025-06-02

**Authors:** Ruanxian Dai, Zhantao Duan, Bin Han, Guobing Chen, Fuping Wang, Zhuange Shi, Xian Zhou, Haifei Song, Li Ma, Qiang Meng

**Affiliations:** ^1^Faculty of Life Science and Technology, Kunming University of Science and Technology, Kunming, China; ^2^Department of Emergency, The First People’s Hospital of Yunnan Province, Kunming, China; ^3^The First Affiliated Hospital of Kunming Medical University, Kunming, China; ^4^Department of Neurology, The First People’s Hospital of Yunnan Province, Kunming, China

**Keywords:** ibotenic acid, neurotoxic mushroom poisoning, neurotransmitters, metabolomics, toxin

## Abstract

**Background:**

Mushroom poisoning represents a significant food safety issue globally, particularly neurotoxic mushroom poisoning, which raises considerable concern due to its potential to induce central nervous system symptoms. Ibotenic acid is identified as the primary neurotoxin associated with this form of poisoning; however, the underlying mechanisms of its neurotoxicity remain poorly understood.

**Objective:**

This study aims to systematically evaluate the effects of ibotenic acid exposure across three consecutive key time points, from intoxication to recovery, on neurotransmitters related to the GABA/Glutamic-Acid, dopaminergic, serotonergic, and cholinergic systems in five brain regions: the cerebral cortex, hippocampus, striatum, brain stem, and cerebellum.

**Methods:**

Through behavioral tests, we assessed the effects of ibotenic acid exposure on voluntary activities and learning and memory functions in mice. Additionally, we analyzed the changes in neurotransmitter concentrations across different brain regions using targeted metabolomics.

**Results:**

Behavioral results indicated that the total movement distance and speed in the open field test were significantly reduced, while the resting time was prolonged in the ibotenic acid-exposed group (*P* < 0.0001). The results of targeted metabolomics demonstrated that, compared to the control group, levels of glutamic acid in the hippocampus and brain stem significantly decreased after 4 h of ibotenic acid exposure (*P* < 0.05, *P* < 0.001). Additionally, epinephrine levels in the cerebral cortex decreased at 20 min (*P* < 0.05), while tyrosine levels in the brain stem and cerebellum decreased after 4 h (*P* < 0.05). In the brain stem region, the tryptophan levels in each exposure group decreased significantly compared with the 4-h exposure group (*P* < 0.01), and brain stem choline levels significantly decreased (*P* < 0.05). Conversely, homovanillic acid levels in the brain stem increased (*P* < 0.01).

**Conclusion:**

Preliminary studies have demonstrated that acute exposure to ibotenic acid inhibits motor activity but does not significantly affect learning and memory in mice. Exposure to ibotenic acid induces alterations in GABA/Glutamic-Acid, dopaminergic, serotonergic, and neurotransmitters associated with the cholinergic system in the brains of mice, with the most pronounced changes occurring in the brain stem region, exhibiting time-dependent and region-specific effects. This study offers new insights into the neurotoxic mechanisms of ibotenic acid.

## 1 Introduction

Mushroom poisoning is a global food safety concern and is among China’s leading causes of foodborne illnesses (Cheng et al., 2023; Vaibhav et al., 2023). Notably, neurotoxic mushroom poisoning has garnered considerable attention due to its propensity to induce symptoms affecting the central nervous system. Ibotenic acid is one of the primary neurotoxins implicated in this type of poisoning and is commonly found in species such as *Amanita muscaria* and *Amanita pantherina* (Su et al., 2023). In recent years, incidents of poisoning resulting from the accidental ingestion of mushrooms containing this toxin have become increasingly frequent, posing a serious threat to public health (Meisel et al., 2022; Ordak et al., 2023). Affected individuals often present with neurological symptoms, including hallucinations, ataxia, and seizures, which significantly heighten the risk of accidental injury and mortality (Moss and Hendrickson, 2019; Maung et al., 2023; Soyka, 2023). Clinical treatment primarily consists of nonspecific supportive care, as no specific antidote is available.

Ibotenic acid, structurally akin to glutamic acid, can penetrate the blood-brain barrier (Stebelska, 2013). Once inside the central nervous system, ibotenic acid undergoes decarboxylation to form muscimol, which bears structural similarity to γ-aminobutyric acid (GABA) and acts as a potent agonist of GABAA receptors (Issahaku et al., 2024). Research indicates that ibotenic acid is the precursor to muscimol, the well-tolerated and primary psychoactive compound in *Amanita muscaria* (Obermaier and Müller, 2020).

Glutamic acid and GABA are the brain’s predominant excitatory and inhibitory neurotransmitters essential for maintaining normal nervous system (Zhou and Danbolt, 2014; Li et al., 2021). Disturbances in neurotransmitter levels often lead to brain dysfunction, which is closely associated with various mental disorders and neurodegenerative diseases (Haleem, 2019; Daws et al., 2022; Goodwin et al., 2022; He et al., 2023). Animal studies have demonstrated that exposure to ibotenic acid significantly increases serotonin concentrations in the brains of male mice and rats (König-Bersin et al., 1970). However, research on the effects of ibotenic acid on other key neurotransmitter systems remains limited. Metabolomics, which enables the simultaneous quantification of thousands of metabolites, is instrumental in elucidating mechanisms of toxicity (Alseekh et al., 2021; Markin et al., 2021b). High-performance liquid chromatography-tandem mass spectrometry (HPLC-MS/MS) is among the most widely utilized methods for analyzing specific neurotransmitters and neurotransmission-related components (Kim and Heyman, 2018).

This study utilizes both the open field and Y-maze tests to evaluate the effects of ibotenic acid exposure on spontaneous activity and learning and memory functions in mice. An acute poisoning model was subsequently established at a dosage of 16 mg/kg. Targeted metabolomics analysis was conducted at three consecutive key time points to assess changes in neurotransmitters associated with the GABA/Glutamic-Acid, dopaminergic, serotonergic, and cholinergic systems across five brain regions: the cerebral cortex, hippocampus, striatum, brain stem, and cerebellum. This study aims to systematically characterize the dynamic alterations of neurotransmitters during ibotenic acid intoxication and recovery while also preliminarily elucidating the potential mechanisms underlying its neurotoxicity.

## 2 Materials and methods

### 2.1 Animals

Male Kunming mice, aged 7–8 weeks, were obtained from Kunming Medical University [animal license number: SCXK (Dian) K2020-0004]. The mice were housed in a facility maintaining a 12-h light/dark cycle and were randomly assigned to experimental and control groups. The room temperature was kept at 22 ± 1°C. Before the experiment, the animals could acclimate to their environment for 1 week and had unrestricted access to food and water.

### 2.2 Chemicals and reagents

Ibotenic acid was purchased from APExBIO (Houston, United States) with a purity of 98%. HPLC-grade acetonitrile (ACN) and methanol (MeOH) were purchased from Merck (Darmstadt, Germany). MilliQ water (Millipore, Bradford, United States) was used in all experiments. Neurotransmitter was purchased from Sigma-Aldrich. All standards were purchased from Olchemlm Ltd. (Olomouc, Czech Republic). The standard stock solutions were prepared in MeOH at 1 mg/mL concentration. All stock solutions were stored at –20°C. The stock solutions were diluted with MeOH to work solutions before analysis.

### 2.3 Experiment design

Based on the findings of previous studies and preliminary experiments, a poisoned mouse model was established through the intraperitoneal injection of 16 mg/kg ibotenic acid (König-Bersin et al., 1970). Observations from preliminary experiments indicated that the mice exhibited reduced activity, unresponsiveness, and unsteady gait approximately 20 min after exposure to 16 mg/kg ibotenic acid. With prolonged exposure, around 1 h post-injection, the mice demonstrated signs of lethargy, accompanied by intermittent tremors and convulsions. After 4 h of exposure, the mice gradually returned to normal activity levels. No significant abnormalities were observed in the control group, which received an equivalent volume of normal saline via intraperitoneal injection. Consequently, three consecutive time points were established to assess brain status: 20 min, 1 h, and 4 h post-exposure. To verify the behavioral changes in the mice, behavioral tests were conducted before the experiment, dividing subjects into an experimental group (*n* = 9) and a control group (*n* = 9).

**FIGURE 1:**
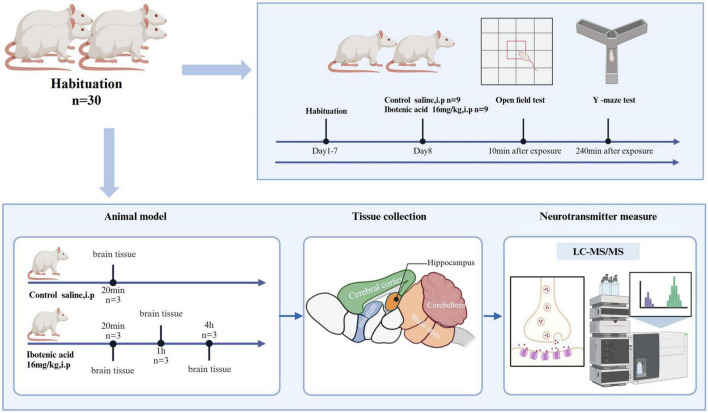
A graphic illustration of the experimental design (created with BioRender.com).

To evaluate the time dependence of neurotransmitter changes following ibotenic acid exposure, we selected the same batch of mice for the experiments. The experiment was randomly divided into four groups: an ibotenic acid exposure group for 20 min (*n* = 3), an exposure group for 1 h (*n* = 3), an exposure group for 4 h (*n* = 3), and a control group (*n* = 3). The exposure groups received an intraperitoneal injection of 1 mg/mL ibotenic acid saline solution, while the control group was injected with an equivalent volume of saline. Mice were euthanized by cervical dislocation, followed by decapitation for tissue collection, and the cerebral cortex, hippocampus, striatum, brain stem, and cerebellum of each mouse were quickly dissected on ice packs. The isolated brain tissue was immediately frozen in liquid nitrogen. White matter was preserved during sampling to study metabolic changes in the original anatomical state. All tissue samples were cryopreserved at -80°C for subsequent analysis. The experimental design is illustrated in Figure 1.

### 2.4 Behavioral tests

The experiments were conducted in the following sequence: the open field test was performed 10 min after ibotenic acid exposure, followed by the Y-maze test conducted 4 h post-exposure. Before testing, the animals were removed from the animal room and allowed to acclimate in their cages for 1 h. While recording the experimental session, the operator remained outside the testing room.

The open-field test evaluates voluntary motor activity, general anxiety-like behavior, and exploratory behavior in animals (Zhang et al., 2024). This study specifically examined the voluntary locomotor activities of mice. In the experiment, the mice were placed in a behavioral box measuring 50 × 50 × 40 cm (width × length × height), constructed from white PVC material. Behavioral data were recorded using a camera positioned above the box and connected to a computer. The base of the box consists of a closed planar area, which is uniformly divided into 25 squares. The central area, comprising nine squares (30 × 30 cm), is designated as the central area, while the remainder constitutes the peripheral area. Following a 10-min exposure to ibotenic acid, the mice were allowed to move freely within the box for an additional 10 min, during which their total movement distance, time spent in the central area, and immobility time were documented. After each trial, the bottom and sides of the PVC box were cleaned with 75% medical alcohol and thoroughly dried using a hair dryer.

The exploratory Y-maze experimental model is employed to assess spatial recognition memory in rodents (Fu et al., 2017). The Y-maze is constructed from black plates and consists of three arms, with an angle of 120 degrees between adjacent arms. Each arm measures 30 × 8 × 15 cm (length × width × height). The three arms are randomly designated as the start arm, the novel arm, and the other arm. The starting arm is always accessible, and the mouse is placed at one end of this arm, allowing for free exploration; the novel arm remains closed during the training period and is opened during the testing period, while the other arms are consistently kept open. Bedding is laid at the bottom of the maze, and the bedding in the three arms is evenly mixed between two experiments to mitigate the influence of odor on the results. Mice are allowed to resume normal activities for 4 h after exposure to ibotenic acid before the experiments commence. The experimental procedure is divided into training and test periods, with a 1-h interval between the two to evaluate spatial recognition memory. The training period lasts 10 min, during which the novel arm is blocked, allowing each mouse to explore freely only in the starting and other arms. After 1 h, the test period begins; the barrier of the novel arm is removed, and the mouse is placed back at the head of the start arm, allowing it to explore the maze for 5 min freely. All experimental procedures are recorded using video equipment, and the videos are analyzed to quantify the number, time, and percentage of distance that the mice spend entering the novel arm compared to the other arms.

### 2.5 Sample preparation and extraction

After the sample was thawed and smashed, 0.05 g was mixed with 500 μL of 70% methanol/water. The sample was vortexed for 3 min under 2,500 r/min and centrifuged at 12,000 r/min for 10 min at 4°C. A 300 μL of supernatant was taken into a new centrifuge tube and placed in a –20°C refrigerator for 30 min; then, the supernatant was centrifuged again at 12,000 r/min for 10 min at 4°C. After centrifugation, 200 μL of supernatant was transferred for further LC-MS analysis.

### 2.6 Ultra performance liquid chromatography-mass spectrometry

Chromatographic separation was conducted using the ExionLC™ AD UPLC system coupled to the QTRAP^®^ 6500+ Triple Quad tandem mass spectrometer equipped with electrospray ionization source (SCIEX, Netherlands) and controlled by Analyst 1.6 software (AB Sciex). The liquid chromatograph was equipped with a Waters ACQUITY UPLC HSS T3 C18 (100 mm × 2.1 mm, 1.8 μm). The column oven temperature was set at 40°C. A gradient elution was used (eluent solution A—0.1% formic acid in water, eluent solution B—0.1% formic acid in acetonitrile), which is described in Supplementary Table S1. ESI-MS/MS Conditions: AB 6500+ QTRAP^®^ LC-MS/MS System, equipped with an ESI Turbo Ion-Spray interface, operates in positive and negative ion modes. The ESI source operation parameters were as follows: ion source, turbo spray; source temperature 550°C; ion spray voltage (IS) 5,500 V (Positive), -4500 V (Negative); curtain gas (CUR) were set at 35.0 psi; DP and CE for individual MRM transitions was done with further DP and CE optimization. A specific set of MRM transitions was monitored for each period according to the neurotransmitters eluted within this period. The MultiQuant 3.0.3 software was utilized to process the mass spectrometry data. The chromatographic peaks detected in various analyte samples were integrated and calibrated by referencing the standard’s retention time and peak shape information to ensure accurate qualitative and quantitative results. Metabolomic sequencing was conducted by Wuhan Metwell Biotechnology Co., Ltd. (Wuhan, China).

### 2.7 Statistical analysis

Metabolomics data was presented as ng/g. Statistical analyses and data visualizations were performed using GraphPad Prism (version 8.3.0). The results were presented as mean ± SEM. The normality of the data was evaluated using the Shapiro-Wilk test. For comparisons involving more than two groups, a one-way analysis of variance (ANOVA) was conducted, followed by Tukey’s *post-hoc* test for multiple comparisons. In cases where the data did not satisfy the normality assumption, non-parametric alternatives were utilized; the Kruskal-Wallis test with Dunn’s *post-hoc* test was employed as an alternative approach. An unpaired *t*-test was utilized for pairwise comparisons between the two groups. A *p*-value of < 0.05 was considered statistically significant.

## 3 Results

### 3.1 Behavior characteristic

**FIGURE 2:**
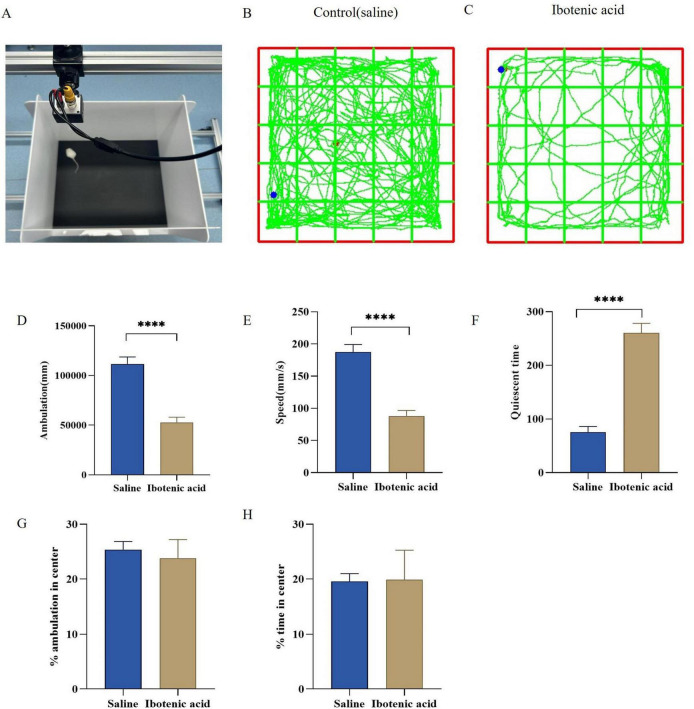
**(A–C)** Trajectory map of the open field test in mice. **(D,E)** Exposure to ibotenic acid significantly decreased both the total ambulation and speed of the mice (*P* < 0.0001), **(F)** quiescent time increase (*P* < 0.0001). **(G,H)** ibotenic acid exposure did not significantly affect the percentage of ambulation in the center and time in the central area (*P* > 0.05). Data are expressed as mean ± SEM. Statistical analysis was done by unpaired *t*-test (****P < 0.0001). *P* < 0.05 was considered a significant difference across groups.

The trajectory of the open-field experiment is illustrated in Figures 2A–C. Compared to the saline group, exposure to ibotenic acid significantly decreased both the total ambulation and speed of the mice (*P* < 0.0001) (Figures 2D,E) while also significantly increasing their quiescent time (*P* < 0.0001) (Figure 2F). Furthermore, ibotenic acid exposure did not significantly affect the percentage of ambulation in the center and time in the central area (Figures 2G,H). These results indicate that ibotenic acid can lead to a reduction in spontaneous activity in mice; however, short-term acute exposure does not appear to induce behavioral abnormalities associated with anxiety or depression.

**FIGURE 3:**
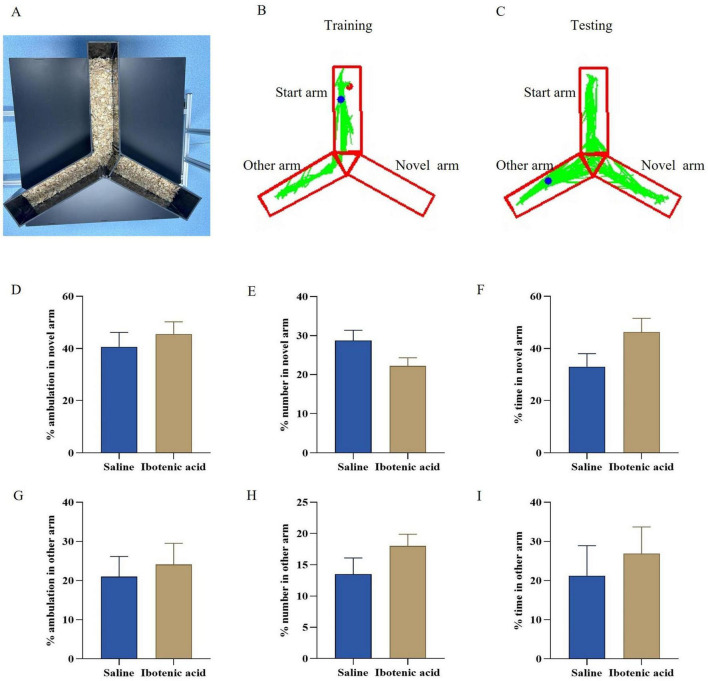
**(A–C)** Schematic illustration of the 2-trial Y-maze tests. **(D–I)** Exposure to ibotenic acid did not significantly impact the percentage of ambulation, the percentage of number, and the percentage of time in the mice’s novel arm and other arm compared to saline controls. Data are expressed as mean ± SEM. Statistical analysis was done by unpaired *t*-test. *P* < 0.05 was considered significant differences across groups.

Figures 3A–C present a schematic diagram of two Y-maze tests. In behavioral assessments involving ibotenic acid-intoxicated mice, we observed that during the recovery period, exposure to ibotenic acid did not significantly impact the percentage of ambulation, the percentage of number, and the percentage of time in the novel arm and other arm of the mice when compared to saline controls (Figures 3D–I). These results suggest that short-term acute exposure to ibotenic acid did not significantly affect mice’s learning and memory capabilities during the recovery period.

### 3.2 Stability of metabolomics methods

**FIGURE 4:**
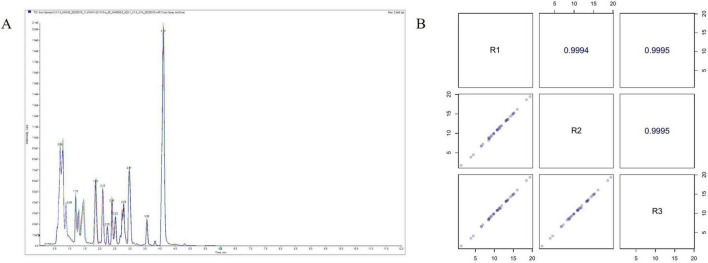
Sample QC analysis. **(A)** Total ion flow chart (TIC) overlap. **(B)** QC sample correlation analysis.

In this study, we selected a mixed solution as a quality control (QC) sample. We compared the total ion current (TIC) analyzed by mass spectrometry of the same QC sample to evaluate the instrument’s performance throughout the detection process. The figure below illustrates the TIC curve of the same QC sample detected by mass spectrometry, demonstrating a significant overlap (Figure 4A). By conducting Pearson correlation analysis on the QC samples, we obtained correlation coefficients of 0.9994, 0.9995, and 0.9995, respectively, indicating that the entire testing process is highly consistent and stable (Figure 4B). The quality control analysis results further confirm the instrument’s stability and the method’s reliability, thereby ensuring the quality of the data.

### 3.3 Neurotransmitter metabolome response

Thirteen significant neurotransmitters and their associated metabolites were quantified. The 13 brain neurotransmitters and metabolites were classified into four primary signaling pathways: the GABA/Glutamic-Acid signaling pathway (including GABA, Glutamic-Acid, and glutamine), the dopamine signaling pathway (comprising tyrosine, epinephrine, 3-hydroxytyramine, and homovanillic acid), the serotonin signaling pathway (which includes serotonin, tryptophan, 5-hydroxyindoleacetic acid, and 5-hydroxytryptophan), and the cholinergic signaling pathway (featuring acetylcholine and choline). Detailed descriptions of neurotransmitters across different brain regions at various times of ibotenic acid exposure were provided in Supplementary Tables S2–S6.

#### 3.3.1 GABA/glutamic-acid pathway

**FIGURE 5:**
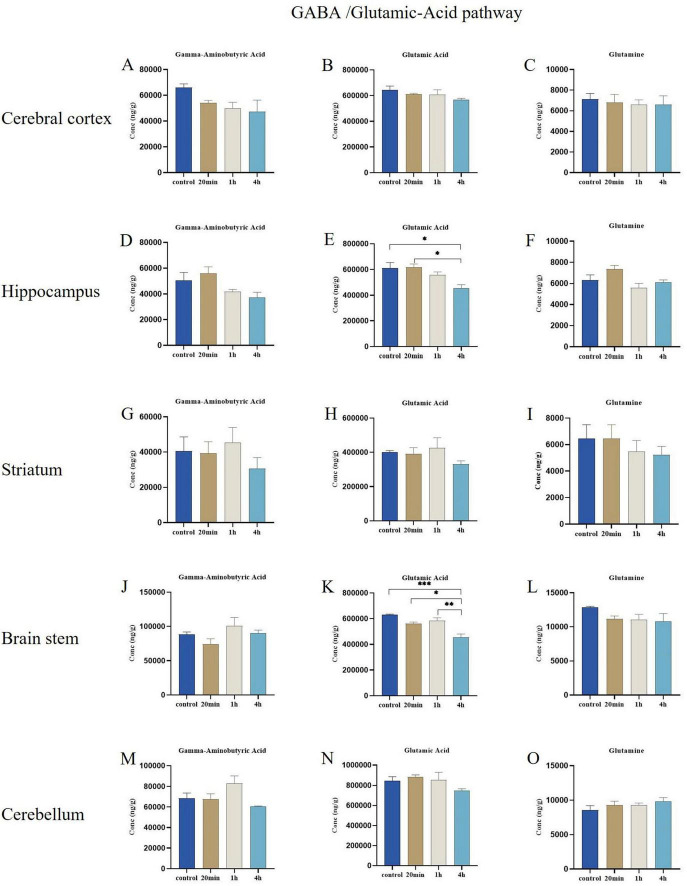
Effects of ibotenic acid exposure on GABA/glutamic-acid pathway-related neurotransmitters in different brain regions of mice in different time groups. **(A–C,G–I,M–O)** No statistically significant differences were observed in the changes of GABA/ Glutamic-Acid pathway-related metabolite concentrations across the cerebral cortex, striatum, and cerebellum regions. **(D,F,J,L)** The concentration changes of GABA and glutamine in the hippocampus region and brain stem regions did not reach statistically significant levels. **(E)** 4-h after exposure to ibotenic acid, the concentration of glutamic-acid in the hippocampus was significantly reduced compared to the control and 20-min exposure groups (*p* < 0.05). **(K)** The concentration of glutamic-acid in the brain stem was decreased in comparison to the control, 20-min exposure, and 1-h exposure groups (*p* < 0.001, *p* < 0.05, and *p* < 0.01, respectively). Concentrations were normalized. Data are expressed as mean ± SEM. Statistical analysis was carried out by one-way analysis of variance (ANOVA) followed by Tukey’s *post-hoc* test for multiple comparisons (*P < 0.05, **P < 0.01, ***P < 0.001).

The results indicated that GABA and glutamic acid levels were highest in distinct brain regions. No statistically significant differences were observed in the changes of GABA/Glutamic-Acid pathway-related metabolite concentrations across the cerebral cortex, striatum, and cerebellum regions from the toxic response to recovery (Figures 5A–C, G–I, M–O), additionally, the concentration changes of GABA and glutamine in the hippocampus region and brain stem regions also did not reach statistically significant levels (Figures 5D, F, J, L). However, 4 h after exposure to ibotenic acid, the concentration of glutamic acid in the hippocampus was significantly reduced compared to the control and 20-min exposure groups (*p* < 0.05) (Figure 5E). Additionally, the concentration of glutamic acid in the brain stem was decreased in comparison to the control, 20-min exposure, and 1-h exposure groups (*p* < 0.001, *p* < 0.05, and *p* < 0.01, respectively) (Figure 5K).

#### 3.3.2 Dopaminergic pathway

**FIGURE 6:**
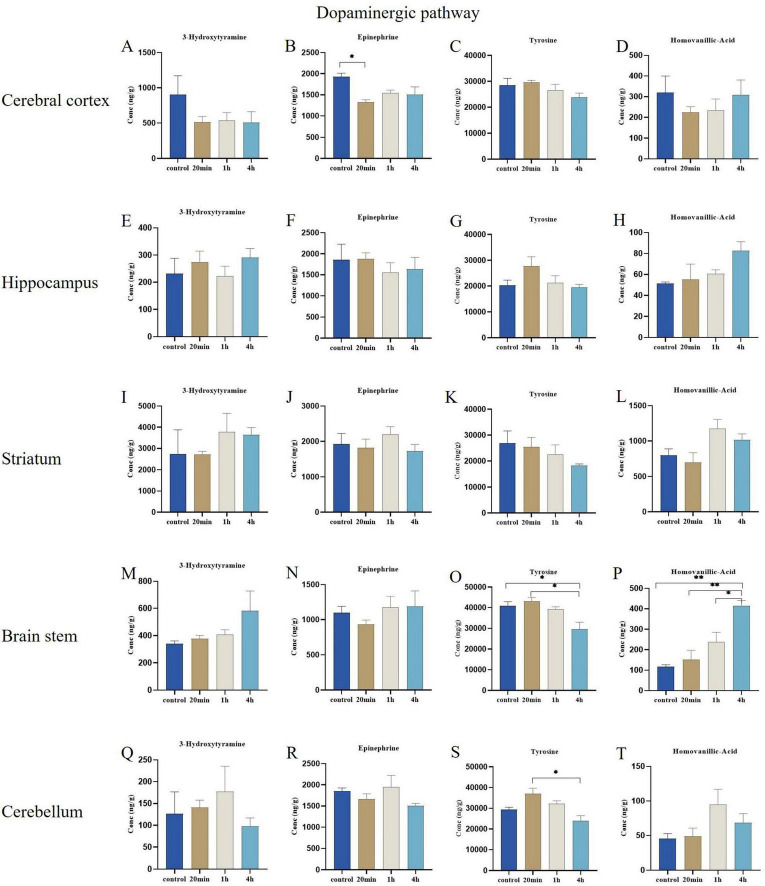
Effects of ibotenic acid exposure on Dopaminergic pathway-related neurotransmitters in different brain regions of mice in different time groups. **(A,C,D)** The concentration changes of 3-hydroxytyramine, tyrosine, and homovanillic acid did not reach statistically significant levels in the cerebral cortex. **(B)** At 20 min post-exposure, the concentration of epinephrine in the cerebral cortex was significantly reduced compared to the control group (*p* < 0.05). **(E–L)** In the hippocampus and striatum regions, no statistically significant differences were observed in the changes in metabolite concentrations associated with the dopaminergic pathway. **(M,N)** The concentrations of 3-hydroxytyramine and epinephrine in the brain stem region were not statistically significant. **(O)** At 4 hours post-exposure, tyrosine concentrations were decreased in the brain stem region relative to both the control and 20-minute exposure groups (*p* < 0.05), **(P)** At 4 hours post-exposure, the concentration of homovanillic acid in the brain stem region was significantly elevated (*p* < 0.01, *p* < 0.01, and *p* < 0.05) compared to the control, 20-minute exposure and 1-hour exposure groups, respectively. **(Q,R,T)** The concentrations of 3-hydroxytyramine, epinephrine, and homovanillic acid did not show statistically significant changes in the cerebellum region. **(S)** At 4 hours after exposure, tyrosine concentrations in the cerebellum region showed a significant decrease (*p* < 0.05) when compared to the 20-minute exposure group. Concentrations were normalized. Data are expressed as mean ± SEM. Statistical analysis was carried out by one-way analysis of variance (ANOVA) followed by Tukey’s *post-hoc* test for multiple comparisons (*P < 0.05, **P < 0.01).

In the hippocampus and striatum regions, no statistically significant differences were observed in the changes in metabolite concentrations associated with the dopaminergic pathway (Figures 6E–L). At 20 min post-exposure, the concentration of epinephrine in the cerebral cortex was significantly reduced compared to the control group (*p* < 0.05) (Figure 6B), additionally, the concentration changes of 3-hydroxytyramine, tyrosine, and homovanillic acid also did not reach statistically significant levels (Figures 6A, C, D). Furthermore, at 4 h post-exposure, tyrosine concentrations were decreased in the brain stem region relative to both the control and 20-min exposure groups (*p* < 0.05) (Figure 6O), changes in 3-hydroxytyramine and epinephrine concentrations were not statistically significant (Figures 6M, N). Similarly, at 4 hours after exposure, tyrosine concentrations in the cerebellar region showed a significant decrease (*p* < 0.05) when compared to the 20-minute exposure group (Figure 6S). However, the concentrations of 3-hydroxytyramine, epinephrine, and homovanillic acid did not exhibit statistically significant changes (Figures 6Q, R, T). In contrast, at 4 h post-exposure, the concentration of homovanillic acid in the brain stem region was significantly elevated (*p* < 0.01, *p* < 0.01, and *p* < 0.05) compared to the control, 20-min exposure, and 1-h exposure groups, respectively (Figure 6P).

#### 3.3.3 Serotonin pathway

**FIGURE 7:**
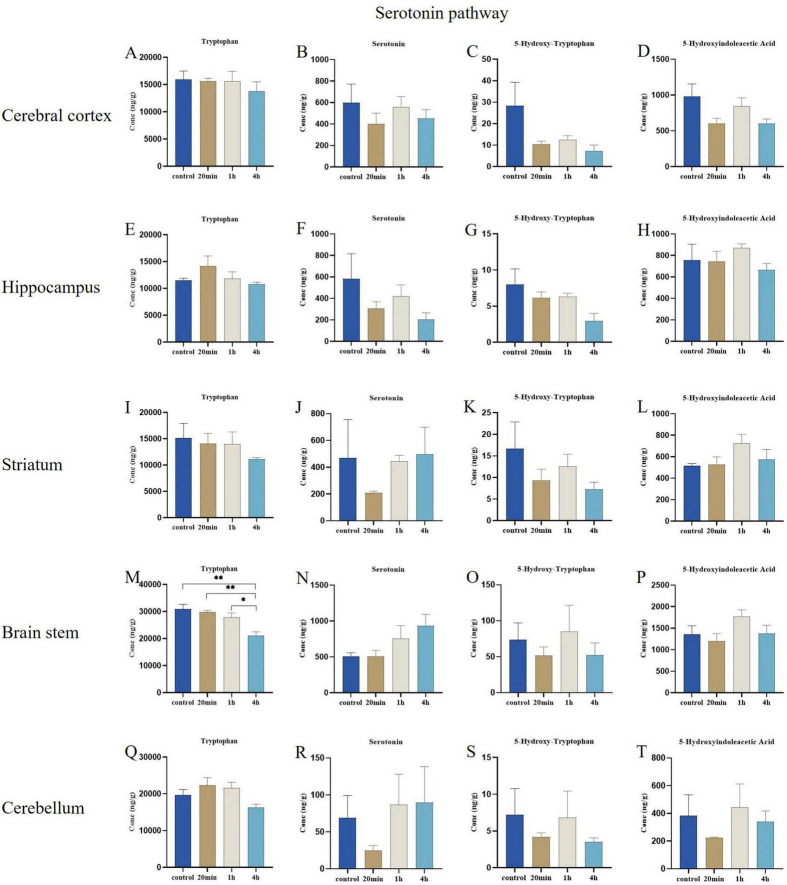
Effects of ibotenic acid exposure on Serotonin pathway-related neurotransmitters in different brain regions of mice in different time groups. **(A–L,Q–T)** There were no significant differences in the concentrations of metabolites associated with the serotonin signaling pathway across the cerebral cortex, hippocampus, striatum, and cerebellum regions. **(M)** The observation was a decrease in tryptophan concentration in the brain stem region at 4 hours post-exposure compared to the control, 20-minute exposure, and 1-hour exposure groups (*p* < 0.01, *p* < 0.01, and *p* < 0.05, respectively). **(N,O,P)** The concentration changes of serotonin, 5-hydroxyindoleacetic acid, and 5-hydroxytryptophan did not achieve statistically significant levels in the brain stem region. Concentrations were normalized. Data are expressed as mean ± SEM. Statistical analysis was carried out by one-way analysis of variance (ANOVA) followed by Tukey’s *post-hoc* test for multiple comparisons. *P* < 0.05 was considered significant differences across groups (*P < 0.05, **P < 0.01).

There were no significant differences in the concentrations of metabolites related to the serotonin signaling pathway across the cerebral cortex, hippocampus, striatum, and cerebellum regions (Figures 7A–L, Q–T). The only notable observation was a decrease in tryptophan concentration in the brain stem region at 4 h post-exposure compared to the control, 20-min exposure, and 1-h exposure groups (*p* < 0.01, *p* < 0.01, and *p* < 0.05, respectively) (Figure 7M), along with a temporal trend indicating higher serotonin concentrations in the brain stem region (Figure 7N), additionally, the concentration changes of 5-hydroxyindoleacetic acid, and 5-hydroxytryptophan also did not reach statistically significant levels (Figures 7O, P).

#### 3.3.4 Cholinergic pathway

**FIGURE 8:**
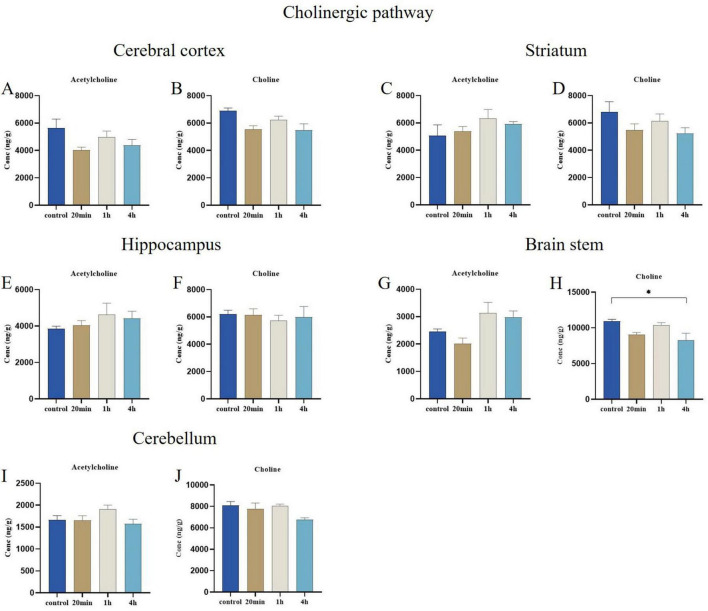
Effects of ibotenic acid exposure on Cholinergic pathway-related neurotransmitters in different brain regions of mice in different time groups. **(A–F,I,J)** No significant differences were observed in the concentrations of metabolites associated with cholinergic signaling pathways across the cerebral cortex, hippocampus, striatum, and cerebellum regions. **(H)** Choline concentrations in the brain stem region was significantly reduced (*p* < 0.05) compared to controls at 4 hours post-exposure. **(G)** The concentration changes of acetylcholine did not reach statistically significant levels in the brain stem region. Concentrations were normalized. Data are expressed as mean ± SEM. Statistical analysis was carried out by one-way analysis of variance (ANOVA) followed by Tukey’s *post-hoc* test for multiple comparisons. *P* < 0.05 was considered significant differences across groups (*P < 0.05).

No significant differences were observed in the concentrations of metabolites associated with cholinergic signaling pathways in the cerebral cortex, hippocampus, striatum, and cerebellum regions (Figures 8A–F, I, J). However, choline concentrations in the brain stem region was significantly reduced (*p* < 0.05) compared to controls at 4 h post-exposure (Figure 8H), additionally, the concentration changes of acetylcholine also did not reach statistically significant levels (Figure 8G).

**FIGURE 9:**
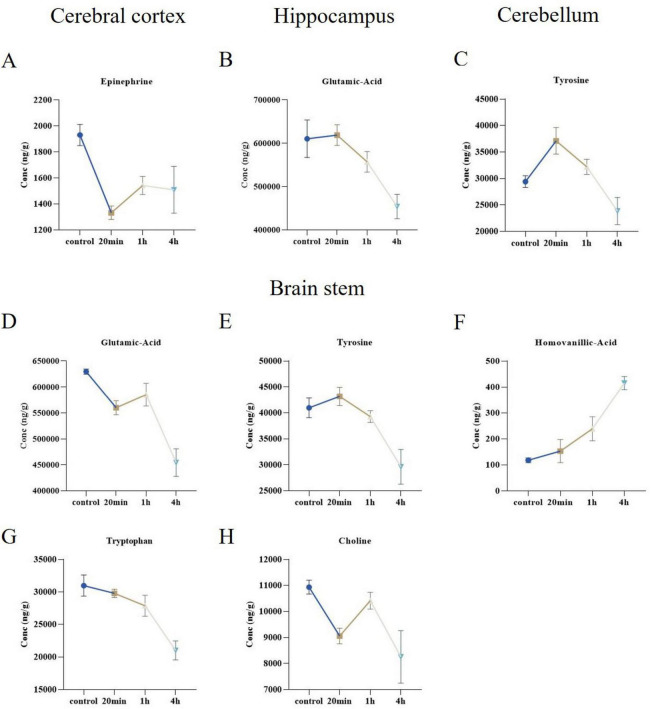
Ibotenic acid exposure at different times showed significant changes in neurotransmitter in different brain regions. **(A–E,G,H)** The concentrations of epinephrine, glutamic-acid, tyrosine, tryptophan, and choline generally displayed a downward trend. **(F)** The concentration of homovanillic acid exhibited a significant upward trend. Data are expressed as mean ± SEM.

**FIGURE 10:**
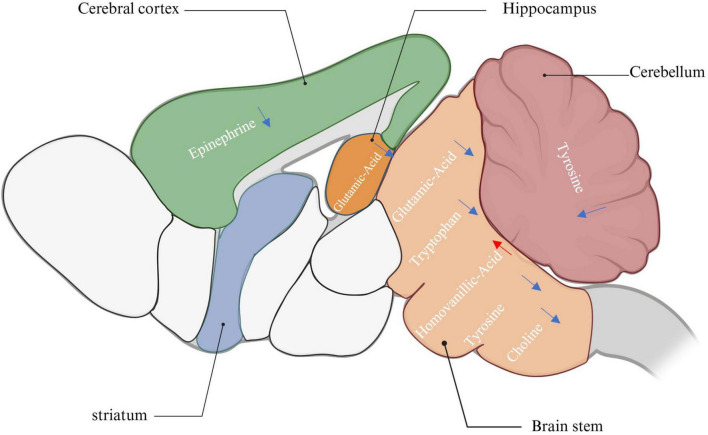
The effect of ibotenic acid on the concentration of neurotransmitters in various brain regions. The blue arrow represent decreased concentrations of neurotransmitters. The red arrow represent increased concentrations of neurotransmitters (created with BioRender.com).

In studies examining the temporal correlation of neurotransmitters across various brain regions in mice, we observed notable trends in the concentrations of multiple neurotransmitters over time. Specifically, the homovanillic acid concentration exhibited a significant upward trend (Figure 9F). In contrast, the concentrations of glutamic acid, tyrosine, tryptophan, epinephrine, and choline generally trend downward (Figures 9A–E, G, H). Further analysis indicated that the brain stem was the region most significantly affected by changes in neurotransmitter levels, as detailed in Figure 10.

## 4 Discussion

In this study, we assessed the effects of ibotenic acid exposure on spontaneous activity and learning memory in mice through behavioral tests. We analyzed the changes in neurotransmitters across different brain regions using targeted metabolomics. The results indicated that acute exposure significantly inhibited the spontaneous activities of the mice; however, their learning and memory abilities were not significantly affected. Neurotransmitter analyses demonstrated that ibotenic acid-induced time-dependent and region-dependent changes in GABA/Glutamic-Acid, dopaminergic, serotonergic, and related cholinergic system transmitters in various brain regions. These findings provide preliminary insights into the mechanisms underlying its neurotoxicity.

Studies have demonstrated that ibotenic acid exerts a more pronounced behavioral impact on male mice than female mice (Sasaki et al., 2021). Sex hormones, particularly estradiol, are known to have protective effects that mitigate neurotoxicity induced by excitotoxins (Baron et al., 2013). Consequently, male mice exhibit greater susceptibility to ibotenic acid-induced neurotoxicity than their female counterparts. This difference may be attributed to the higher levels of sex hormones present in female mice. Male mice were chosen as the experimental subjects for this study to assess the effects of ibotenic acid exposure on behavioral changes in mice more accurately.

Local injection of ibotenic acid has been demonstrated to directly damage hippocampal neurons and significantly impair spatial and working memory, making it a commonly utilized method for constructing models of neurodegenerative diseases, such as Alzheimer’s disease (Liu et al., 2022). In this study, ibotenic acid was administered via intraperitoneal injection, and the results of the Y-maze test indicated that learning and memory were not significantly affected during the behavioral recovery period, which aligns with clinical observations (Vendramin and Brvar, 2014).

In this study, GABA and glutamic acid exhibited the highest levels in various brain regions compared to the control group, underscoring their fundamental roles as the primary inhibitory and excitatory neurotransmitters in neural signal transmission. We observed that glutamic acid concentrations were significantly reduced following exposure to ibotenic acid, particularly in the hippocampus and brain stem regions. Conversely, the concentrations of GABA/Glutamic-Acid pathway-related metabolites in the cerebral cortex, striatum, and cerebellum did not display significant differences throughout the entire process from toxic reaction to recovery, suggesting that these brain regions maintain a stable response to toxicological stimuli. The GABA/glutamate pathway demonstrates a high degree of stability. Previous studies have indicated that the decarboxylation of ibotenic acid can yield muscimol, which binds with high affinity to inhibitory GABA receptors (Issahaku et al., 2024; Rivera-Illanes and Recabarren-Gajardo, 2024). Following the ingestion of ibotenic acid, it manifests both stimulant and inhibitory effects, leading to alternating experiences of euphoria, anxiety, confusion, delusions, and hallucinations (Vahter et al., 2007), which align with clinically observed symptoms (Maung et al., 2023). Behavioral experiments confirmed the significant effects of ibotenic acid exposure on the spontaneous activities of mice. Based on the behavioral performance of the mice and the metabolic processing of ibotenic acid in the brain, we hypothesize that the decrease in glutamic acid results from increased conversion to GABA, leading to an excitatory-inhibitory imbalance in the brain, further corroborating previous research. The imbalance between excitatory and inhibitory neurotransmitters is linked to various neurological and psychiatric disorders (Sohal and Rubenstein, 2019; Xie et al., 2022). The physiological mechanisms underlying this phenomenon warrant further investigation.

In the present study, we observed a significant decrease in cerebral cortical epinephrine concentrations during the early stages following exposure to ibotenic acid. Epinephrine is widely distributed across organisms and is crucial in brain and nerve signaling (Cheng et al., 2021). The decrease in epinephrine concentration was most pronounced at the initial exposure stage, subsequently showing a gradual increase, which exhibited a “biphasic” response pattern. This pattern suggests that the body’s stress response regulatory mechanism is disturbed. Additionally, we noted reductions in tyrosine concentrations within the brain stem and cerebellum regions. Tyrosine serves as the precursor amino acid for the synthesis of monoamines in the dopamine family, and fluctuations in its concentration may correlate with increased expression of tyrosine hydroxylase, the enzyme responsible for catalyzing the conversion of tyrosine (Markin et al., 2021a). The upregulation of this enzyme may contribute to the observed decrease in tyrosine concentration. A decline in tyrosine levels within the brain stem is likely to limit dopamine synthesis, potentially leading to symptoms such as depression, loss of interest, and anhedonia, thereby increasing the risk of depression and other mood disorders. The behavioral manifestations observed in the mice during this study included reduced spontaneous activity, which may be linked to alterations in tyrosine concentration. Homovanillic acid (HVA) concentrations in the brain stem region increased over time. HVA, the principal metabolite of dopamine, is recognized as a central nervous system (CNS) biomarker for dopamine-related diseases (Cardoso et al., 2023). Variations in its concentration reflect the metabolism of dopamine, which may result from toxicological stimulation that triggers dopamine release, potentially bypassing standard regulatory mechanisms—the underlying mechanisms driving changes in homovanillic acid concentrations following exposure warrant further investigation.

In the present study, we observed that exposure to ibotenic acid resulted in a significant decrease in regional tryptophan concentrations in the brain stem, accompanied by an increasing trend in serotonin levels. Tryptophan serves as a precursor to serotonin, and its metabolism has been shown to impact serotonin levels (Gao et al., 2020). When tryptophan concentrations decrease, serotonin synthesis is also reduced. In our study, brain stem tryptophan concentration significantly decreased after 4 hours of exposure; meanwhile, serotonin concentration, although not exhibiting significant differences compared to other brain regions, may exhibit potential changes over time. This finding contradicts previous studies (König-Bersin et al., 1970). Warranting further exploration of the specific mechanisms involved.

Our results indicate that choline concentrations were significantly reduced in mice’s brain stem regions following 4 h of exposure to ibotenic acid. Given that choline serves as the precursor for acetylcholine, this decrease in concentration directly impacts the synthesis of acetylcholine. Prior studies have established a close relationship between choline and brain function, particularly concerning memory and learning capabilities (Téglás et al., 2019). Acetylcholine is crucial for forming spatial memory in the hippocampus, and diminished neurotransmitter levels are closely associated with memory-related disorders, such as Alzheimer’s (Yang et al., 2024). However, Y-maze behavioral experiments conducted after the return to normal activities revealed that ibotenic acid exposure did not significantly affect learning and memory. We speculate that despite reducing choline concentration in specific brain regions, other related metabolic pathways may compensate to maintain acetylcholine levels. Alternatively, the learning and memory assessed in the Y-maze experiment may depend more on other brain regions, such as the hippocampus or prefrontal cortex, which were not significantly impacted. The timing of our measurements may not fully capture these dynamic changes. Future research focusing on the dynamic alterations in choline metabolism will be essential.

*Amanita muscaria* is widely recognized for its hallucinogenic metabolites, ibotenic acid, and muscimol. Recently, there has been a growing interest in exploring the pharmacological potential of *Amanita muscaria* (Zavadinack et al., 2021; Dushkov et al., 2023). The manufacturers that convert ibotenic acid to muscimol in extracts and isolates are considering mitigating safety risks by offering products focusing on *Amanita muscaria’s* therapeutic potential to help with stress, pain, and sleep. This phenomenon not only reflects the public’s continuous concern about hallucinogenic mushrooms but also triggers thoughts on the boundary between traditional toxicology and emerging pharmacopeias.

Although this study primarily investigates the acute neurotoxic effects of ibotenic acid, the implications of chronic or low-dose repeated exposure warrant attention. Previous research indicates that environmental exposure to fungal neurotoxins may be linked to the onset of amyotrophic lateral sclerosis (ALS) (Lagrange et al., 2022). Therefore, future research should prioritize assessing potential risks associated with ibotenic acid in populations regularly consuming mushrooms.

This is the first time a correlation study has been conducted to apply targeted metabolomics to assess neurotransmitter changes across multiple brain regions following exposure to ibotenic acid in mice. Several limitations warrant discussion. First, only five brain regions were analyzed in this study. Second, using only male mice in early adulthood restricts the ability to investigate variations in ibotenic acid metabolism across different ages, hormonal states, and sexes. Third, the absence of a follow-up period limits our capacity to assess time dependence accurately. Future studies could be expanded to encompass a broader range of brain regions, include male and female mice of varying ages and conditions, and investigate the long-term effects of a dose-response relationship to validate these findings further. It is important to note that the experimental design included only three replicates; thus, the results should be interpreted as preliminary.

## 5 Conclusion

In summary, Preliminary studies have demonstrated that acute exposure to ibotenic acid significantly inhibits motor activity; however, it does not significantly affect learning and memory in mice. Exposure to ibotenic acid induces alterations in GABA/ Glutamic-Acid, dopaminergic, serotonergic, and neurotransmitters associated with the cholinergic system in the brains of mice, with the most pronounced changes occurring in the brain stem region, exhibiting time-dependent and region-specific effects. This study offers new insights into the neurotoxic mechanisms of ibotenic acid.

## Data Availability

The original contributions presented in this study are included in this article/Supplementary material, further inquiries can be directed to the corresponding author.
