# Supplementary material for: Preliminary study on the time-correlation changes in brain neurotransmitters of mice exposed to mushroom toxin ibotenic acid
Source: Front Neurosci. 2025 Jun 2;19:1561291. doi: 10.3389/fnins.2025.1561291 (PMC12171373; doi:10.3389/fnins.2025.1561291)
Supplement: Supplementary file 1 [file Table_1.docx]

Table1 Column mobile phase gradient condition.

| Time(min) | Flow rate (mL/min) | A(%) | B(%) |
| --- | --- | --- | --- |
| 0 | 0.35 | 95 | 5 |
| 8 | 0.35 | 5 | 95 |
| 9.5 | 0.35 | 5 | 95 |
| 9.6 | 0.35 | 5 | 95 |
| 12 | 0.35 | 95 | 5 |
