# Supplementary material for: Preliminary study on the time-correlation changes in brain neurotransmitters of mice exposed to mushroom toxin ibotenic acid
Source: Front Neurosci. 2025 Jun 2;19:1561291. doi: 10.3389/fnins.2025.1561291 (PMC12171373; doi:10.3389/fnins.2025.1561291)
Supplement: Supplementary file 2 [file Table_2.docx]

| Table 2.The Concentrations of Neurotransmitters in the Cerebral cortex of Mice | | | | | |
| --- | --- | --- | --- | --- | --- |
| **Neurotransmitter system and matabolite pathways** | **Role** | **Brain tissue content［ng/g，M±SD］** | | | |
|  |  | **Cerebral cortex** | | | |
|  |  | **control** | **20min** | **1h** | **4h** |
| **GABA/Glutamic-Acid**  **pathway** | |  |  |  |  |
| GABA | Neurotransmitter | 65963.7±5096.6 | 54231±3083.9 | 49853.5±8188.6 | 47408.5±15437 |
| Glutamic-Acid | Neurotransmitter | 645089.3±50850.7 | 610863.2±11702.8 | 607646±64995.4 | 566451.9±22755.3 |
| Glutamine | Precursor | 7141.2±918.7 | 6822.3±1306.8 | 6584.4±818.5 | 6606±1456.7 |
| **Dopaminergic pathway** |  |  |  |  |  |
| Tyrosine | Precursor | 28517.2±4603.3 | 29601.9±1284.9 | 26610.5±3897.7 | 23864.4±2720.3 |
| Epinephrine | Neurotransmitter | 1930.6±141.2 | 1331.7±89.4 | 1542.3±120.7 | 1508.7±311.3 |
| 3-Hydroxytyramine | Neurotransmitter | 905.9±458.3 | 512.8±140.2 | 535.2±196.7 | 507.2±265.2 |
| Homovanillic-Acid | Metabolite | 320.3±138.1 | 225.1±46.2 | 234.6±94.2 | 308.7±124.9 |
| **Serotonin pathway** |  |  |  |  |  |
| Tryptophan | Precursor | 15901.4±2722.9 | 15654±865.6 | 15613.2±3179.6 | 13800.3±2948.6 |
| 5-Hydroxyindoleacetic-Acid | Metabolite | 982.8±298.3 | 604.3±122.2 | 842±207.2 | 604.7±104.4 |
| Serotonin | Neurotransmitter | 597.9±301.3 | 400.7±175.7 | 559.4±166.9 | 453.5±137.4 |
| 5-Hydroxy-Tryptophan | Precursor | 28.3±18.8 | 10.3±2.5 | 12.5±3.2 | 7.1±4.9 |
| **Cholinergic pathway** |  |  |  |  |  |
| Acetylcholine | Neurotransmitter | 5634.3±1131.5 | 4029.8±373.8 | 4977.3±758.8 | 4391.6±711.4 |
| Choline | Precursor | 6900.9±337.3 | 5541.4±456.3 | 6225.3±485 | 5483.3±798 |
