# Supplementary material for: Preliminary study on the time-correlation changes in brain neurotransmitters of mice exposed to mushroom toxin ibotenic acid
Source: Front Neurosci. 2025 Jun 2;19:1561291. doi: 10.3389/fnins.2025.1561291 (PMC12171373; doi:10.3389/fnins.2025.1561291)
Supplement: Supplementary file 3 [file Table_3.docx]

| Table 3.The Concentrations of Neurotransmitters in the Hippocampus of Mice | | | | | |
| --- | --- | --- | --- | --- | --- |
| **Neurotransmitter**  **system and matabolite pathways** | **Role** | **Brain tissue content［ng/g，M±SD］** | | | |
|  |  | **Hippocampus** | | | |
|  |  | **control** | **20min** | **1h** | **4h** |
| **GABA /Glutamic-Acid**  **pathway** | |  |  |  |  |
| GABA | Neurotransmitter | 50414.8±10920.2 | 55983±8963.3 | 41853.8±3087.6 | 37254.4±7154.3 |
| Glutamic-Acid | Neurotransmitter | 610428.3±75198.5 | 618937.5±41155.2 | 557089±41005 | 453941.8±48744.5 |
| Glutamine | Precursor | 6310.8±873.1 | 7357.1±610.1 | 5601.7±724.1 | 6105.5±396.3 |
| **Dopaminergic pathway** |  |  |  |  |  |
| Tyrosine | Precursor | 20355±3285.7 | 27681.7±6445.4 | 21214.1±4807.1 | 19497.7±1965.7 |
| Epinephrine | Neurotransmitter | 1863.5±631 | 1882.5±249 | 1550.7±420.5 | 1636.2±486.1 |
| 3-Hydroxytyramine | Neurotransmitter | 231±99.5 | 274.4±69.9 | 223±61.6 | 291.3±56.9 |
| Homovanillic-Acid | Metabolite | 51.4±2.5 | 55.1±25.9 | 60.6±6.6 | 82.6±14.8 |
| **Serotonin pathway** |  |  |  |  |  |
| Tryptophan | Precursor | 11537.1±622.8 | 14140.1±3314.7 | 11830.8±2174 | 10798.3±622.3 |
| 5-Hydroxyindoleacetic-Acid | Metabolite | 756.2±258.5 | 743.7±162.5 | 871±64.2 | 664.6±105.8 |
| Serotonin | Neurotransmitter | 583.6±401.5 | 304.8±110.9 | 419.8±184.6 | 205.4±105.9 |
| 5-Hydroxy-Tryptophan | Precursor | 8±3.7 | 6.1±1.3 | 6.3±0.7 | 2.9±1.7 |
| **Cholinergic system** |  |  |  |  |  |
| Acetylcholine | Neurotransmitter | 3845.3±252.9 | 4032.1±466 | 4629.2±1078.4 | 4423.8±679.7 |
| Choline | Precursor | 6217.3±470.9 | 6142.8±778.6 | 5727±674 | 5986.7±1347.2 |
