# Supplementary material for: Preliminary study on the time-correlation changes in brain neurotransmitters of mice exposed to mushroom toxin ibotenic acid
Source: Front Neurosci. 2025 Jun 2;19:1561291. doi: 10.3389/fnins.2025.1561291 (PMC12171373; doi:10.3389/fnins.2025.1561291)
Supplement: Supplementary file 4 [file Table_4.docx]

| Table 4.The Concentrations of Neurotransmitters in the Striatum of Mice | | | | | |
| --- | --- | --- | --- | --- | --- |
| **Neurotransmitter**  **system and matabolite pathways** | **Role** | **Brain tissue content［ng/g，M±SD］** | | | |
|  |  | **Striatum** | | | |
|  |  | **control** | **20min** | **1h** | **4h** |
| **GABA /Glutamic-Acid**  **pathway** | |  |  |  |  |
| GABA | Neurotransmitter | 40680.6±13773.8 | 39447.1±11202 | 45300.3±15075 | 30644.8±10812.3 |
| Glutamic-Acid | Neurotransmitter | 401624.9±14278.9 | 391597.6±62820.9 | 426437.7±103104.1 | 331009.1±33558.9 |
| Glutamine | Precursor | 6449.6±1819.8 | 5853±1348.3 | 5479.5±1444 | 5240.6±1073.5 |
| **Dopaminergic pathway** |  |  |  |  |  |
| Tyrosine | Precursor | 26856.6±8240.3 | 25450.9±6364.4 | 22591.3±6314.3 | 18382.8±935.2 |
| Epinephrine | Neurotransmitter | 1921.5±523.1 | 1821.9±417 | 2197±382.1 | 1726.2±324.1 |
| 3-Hydroxytyramine | Neurotransmitter | 2741.7±1969.1 | 2710.5±269 | 3783.7±1515.2 | 3641±587 |
| Homovanillic-Acid | Metabolite | 798.5±157.1 | 694.9±240.5 | 1173.5±228.3 | 1018±143.6 |
| **Serotonin pathway** |  |  |  |  |  |
| Tryptophan | Precursor | 15162.7±4742.3 | 14051.3±3412.9 | 14008.4±3949.2 | 11096±533.1 |
| 5-Hydroxyindoleacetic-Acid | Metabolite | 517.2±35 | 529±121 | 722.6±146.8 | 572.6±163.1 |
| Serotonin | Neurotransmitter | 469.2±496.2 | 208.6±21.6 | 446.5±73.5 | 496.4±350.9 |
| 5-Hydroxy-Tryptophan | Precursor | 16.7±10.6 | 9.3±4.5 | 12.5±4.8 | 7.3±2.7 |
| **Cholinergic pathway** |  |  |  |  |  |
| Acetylcholine | Neurotransmitter | 5071.3±1366.5 | 5380.6±632.3 | 6334.4±1134.4 | 5939.2±271.7 |
| Choline | Precursor | 6817.4±1280.1 | 5471.5±789.8 | 6153.3±872.7 | 5244.6±692.6 |
