# Supplementary material for: Preliminary study on the time-correlation changes in brain neurotransmitters of mice exposed to mushroom toxin ibotenic acid
Source: Front Neurosci. 2025 Jun 2;19:1561291. doi: 10.3389/fnins.2025.1561291 (PMC12171373; doi:10.3389/fnins.2025.1561291)
Supplement: Supplementary file 5 [file Table_5.docx]

| Table 5.The Concentrations of Neurotransmitters in the Brain stem of Mice | | | | | |
| --- | --- | --- | --- | --- | --- |
| **Neurotransmitter**  **system and matabolite**  **pathways** | **Role** | **Brain tissue content［ng/g，M±SD］** | | | |
|  |  | **Brain stem** | | | |
|  |  | **control** | **20min** | **1h** | **4h** |
| **GABA /Glutamic-Acid**  **pathway** | |  |  |  |  |
| GABA | Neurotransmitter | 88436.5±6172.8 | 74203.2±13526.2 | 100573.8±21464.7 | 90547.2±6900.8 |
| Glutamic-Acid | Neurotransmitter | 629674.8±8783.3 | 559920.5±23488.3 | 585004.3±37887 | 454262.4±45771.8 |
| Glutamine | Precursor | 12841.9±301.4 | 11182.8±736.7 | 11044.1±1393.4 | 10835.7±1963.4 |
| **Dopaminergic pathway** |  |  |  |  |  |
| Tyrosine | Precursor | 29787.4±1107.7 | 29787.4±1107.7 | 27881.8±2799.3 | 21016.1±2516.7 |
| Epinephrine | Neurotransmitter | 1198.6±306.4 | 1198.6±306.4 | 1767.4±277.7 | 1376±330.6 |
| 3-Hydroxytyramine | Neurotransmitter | 506.3±143.9 | 506.3±143.9 | 754.7±310.7 | 930.1±283.1 |
| Homovanillic-Acid | Metabolite | 52±20.2 | 52±20.2 | 85.3±62.5 | 52.4±29 |
| **Serotonin pathway** |  |  |  |  |  |
| Tryptophan | Precursor | 40973.8±3305 | 43168.5±3094.2 | 39275.3±1950.2 | 29620.5±5803.7 |
| 5-Hydroxyindoleacetic-Acid | Metabolite | 1096.9±156.3 | 935±101.4 | 1178.2±270.9 | 1189.5±382 |
| Serotonin | Neurotransmitter | 340.1±37.8 | 378.6±39.7 | 407±62 | 581.6±255.2 |
| 5-Hydroxy-Tryptophan | Precursor | 117.5±15.7 | 152.9±76.7 | 238.6±80.4 | 415.1±43.5 |
| **Cholinergic pathway** |  |  |  |  |  |
| Acetylcholine | Neurotransmitter | 2450.9±171.7 | 2017.3±349.7 | 3135.4±666.5 | 2983.7±395.1 |
| Choline | Precursor | 10934.3±462.8 | 9055.7±518.9 | 10414.8±560.5 | 8255.8±1743.5 |
