# Supplementary material for: Preliminary study on the time-correlation changes in brain neurotransmitters of mice exposed to mushroom toxin ibotenic acid
Source: Front Neurosci. 2025 Jun 2;19:1561291. doi: 10.3389/fnins.2025.1561291 (PMC12171373; doi:10.3389/fnins.2025.1561291)
Supplement: Supplementary file 6 [file Table_6.docx]

| Table 6.The Concentrations of Neurotransmitters in the Cerebellum of Mice | | | | | |
| --- | --- | --- | --- | --- | --- |
| **Neurotransmitter**  **system and matabolite**  **pathways** | **Role** | **Brain tissue content［ng/g，M±SD］** | | | |
|  |  | **Cerebellum** | | | |
|  |  | **control** | **20min** | **1h** | **4h** |
| **GABA /Glutamic-Acid**  **pathway** | |  |  |  |  |
| GABA | Neurotransmitter | 68505.5±8719.4 | 67804.4±8722.3 | 83022±12452.9 | 60745±506.5 |
| Glutamic-Acid | Neurotransmitter | 843655.6±73724.3 | 882853±34044.7 | 852489.5±132685.4 | 747546.6±31822.7 |
| Glutamine | Precursor | 8596.9±1073.6 | 9311.8±955.2 | 9277.1±561.6 | 9813.6±1006.9 |
| **Dopaminergic pathway** |  |  |  |  |  |
| Tyrosine | Precursor | 29410.7±1931.2 | 37118.2±4353.1 | 32197.5±2497 | 23843.2±4438.4 |
| Epinephrine | Neurotransmitter | 1851.3±134.5 | 1660.4±220.1 | 1944.7±483.7 | 1506.7±93.3 |
| 3-Hydroxytyramine | Neurotransmitter | 126.8±86 | 141±28.7 | 177.4±100.4 | 97.6±33.6 |
| Homovanillic-Acid | Metabolite | 45.5±12.4 | 49.2±19.8 | 95.1±37.7 | 68.6±22.6 |
| **Serotonin pathway** |  |  |  |  |  |
| Tryptophan | Precursor | 19647.1±2721.6 | 22311.3±3651.8 | 21640.5±2594 | 16351.7±1427.9 |
| 5-Hydroxyindoleacetic-Acid | Metabolite | 382.1±263.2 | 224.1±8.4 | 445.7±289.2 | 342.3±130.9 |
| Serotonin | Neurotransmitter | 68.9±52.3 | 24.9±11.5 | 86.8±71 | 89.9±83.7 |
| 5-Hydroxy-Tryptophan | Precursor | 7.2±6.1 | 4.2±0.9 | 6.8±6.2 | 3.5±0.9 |
| **Cholinergic pathway** |  |  |  |  |  |
| Acetylcholine | Neurotransmitter | 1665.7±164.3 | 1653±183 | 1907.2±162.9 | 1572.3±186.8 |
| Choline | Precursor | 8115.8±590.7 | 7789.5±916.1 | 8072.2±255.3 | 6759.1±305.9 |
